# A Multi-Step Pathway for the Establishment of Sister Chromatid Cohesion 

**DOI:** 10.1371/journal.pgen.0030012

**Published:** 2007-01-19

**Authors:** Mark Milutinovich, Elçin Ünal, Chris Ward, Robert V Skibbens, Douglas Koshland

**Affiliations:** 1 Howard Hughes Medical Institute, Carnegie Institution, Baltimore, Maryland, United States of America; 2 Department of Embryology, Carnegie Institution, Baltimore, Maryland, United States of America; 3 Department of Biology, Johns Hopkins University, Baltimore, Maryland, United States of America; 4 Ingenuity Program, Baltimore Polytechnic Institute, Baltimore, Maryland, United States of America; 5 Department of Biological Sciences, Lehigh University, Bethlehem, Pennsylvania, United States of America; National Institute of Diabetes and Digestive and Kidney Diseases, United States of America

## Abstract

The cohesion of sister chromatids is mediated by cohesin, a protein complex containing members of the structural maintenance of chromosome (Smc) family. How cohesins tether sister chromatids is not yet understood. Here, we mutate *SMC1*, the gene encoding a cohesin subunit of budding yeast, by random insertion dominant negative mutagenesis to generate alleles that are highly informative for cohesin assembly and function. Cohesins mutated in the Hinge or Loop1 regions of *Smc1* bind chromatin by a mechanism similar to wild-type cohesin, but fail to enrich at cohesin-associated regions (CARs) and pericentric regions. Hence, the Hinge and Loop1 regions of Smc1 are essential for the specific chromatin binding of cohesin. This specific binding and a subsequent Ctf7/Eco1-dependent step are both required for the establishment of cohesion. We propose that a cohesin or cohesin oligomer tethers the sister chromatids through two chromatin-binding events that are regulated spatially by CAR binding and temporally by Ctf7 activation, to ensure cohesins crosslink only sister chromatids.

## Author Summary

Complexes containing members of the structural maintenance of chromosomes (Smc) family regulate higher order chromosome architecture in diverse aspects of DNA metabolism including chromosome condensation, sister chromatid cohesion, DNA repair, and global control of transcription. Smc complexes are thought to regulate higher order chromosome folding by tethering together two strands of chromatin. However, the mechanism of tethering is poorly understood in part because of a poor understanding of the function of the core Smc subunits. To gain insight into the structure and function of Smc subunits, we developed a novel strategy of mutagenesis called random insertion dominant negative (RID), which generates informative alleles with high efficiency and should provide an effective tool to study any multi-subunit complex. Using RID we generated novel alleles of a *Smc* subunit from the cohesin complex. The cohesin complex tethers together newly replicated chromosomes (sister chromatids). The analyses of these RID mutants suggest that the tethering activity of cohesin (and possibly other Smc complexes) is generated by two sequential chromatin-binding events (first the capture of one piece of chromatin followed by the capture of the second piece of chromatin), which are regulated both spatially and temporally. We speculate that the spatial and temporal regulation of cohesin ensures that it tethers together only sister chromatids rather than randomly crosslinking the entire genome.

## Introduction

Proper transmission of eukaryotic chromosomes during cell division requires DNA replication and three other DNA-dependent processes: recombination-dependent DNA repair, sister chromatid cohesion, and chromosome condensation. Each of these diverse processes requires protein complexes containing two members of the highly conserved structural maintenance of chromosomes (Smc) family of proteins [1–3]. Smc complexes likely share a common core activity of chromosome crosslinking, either within a chromosome, as in chromosome condensation, or between chromosomes, for sister chromatid cohesion and recombination-dependent DNA repair. How Smc complexes mediate chromosome crosslinking is unknown.

**Figure 1:**
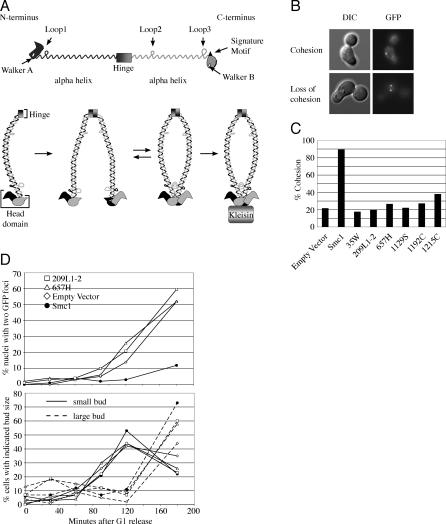
Dominant Negative Insertion Mutants Disrupt Sister Chromatid Cohesion (A) Schematic presents conserved structural and functional features of an Smc protein, folding of an Smc molecule, dimerization with a second Smc protein, and binding of the kleisin subunit (see text). Analysis of sister chromatid cohesion for strains expressing dominant insertion mutants of *SMC1* (B–D). (B) A LacO/LacI-GFP-based system was used to assess sister chromatid cohesion. The yeast strain YMM-202 contains a tandem array of Lac Operators inserted 9.7 kb from one end of Chromosome IV, LacI-GFP, and the temperature sensitive *smc1–2* allele. Under conditions of functional cohesion, sister chromatids are held in close proximity, resulting in a single GFP focus (top). Loss of cohesion allows sister chromatids to precociously separate, resulting in two GFP foci (bottom). (C) YMM-202 harboring minichromosomes with inducible *Smc1* alleles were released from G1 under conditions that inactivate the smc1–2 protein and induce expression of the galactose-regulated allele (see Materials and Methods). Cell cycle progression beyond metaphase was blocked by the addition of nocodazole. Cohesion in the different strains was assessed by counting the number of metaphase-arrested cells with a single GFP focus. (D) YMM-202 harboring minichromosomes with inducible *Smc1* alleles were treated as described in (C), except cells were removed at the indicated intervals after release from G1 and assessed for the appearance of separated sister chromatids (two GFP foci) following DNA replication (upper graph). Cell cycle progression was determined by cell morphology (lower graph). The appearance of two GFP foci occurs with a similar timing for strains with an empty vector or two representative insertion mutants, indicating that these mutants fail to establish cohesion.

Smc molecules are composed of five structural domains (Figure 1A) [4,5]: a globular N-terminal domain containing a Walker A motif, a globular C-terminal domain with Walker B and Signature motifs, two long α-helical domains, and a globular Hinge domain. Smc monomers fold in half at the Hinge domain, allowing the two α-helices to form a long antiparallel coiled-coil domain [6]. This folding juxtaposes the N- and C-terminal globular domains and the Walker A and B motifs, creating an Smc head domain with ATPase activity. Folded Smc monomers resemble a flexible dumbbell, with the Hinge and head domains separated by ∼40 nm of coiled coil [6,7].

Smc complexes are composed of two Smc molecules, a kleisin subunit, and at least one accessory protein [6,8,9]. Smc monomers dimerize primarily through interactions between their Hinge domains [6,10]. The head domains of Smc molecules are also tethered together through the shared binding of a single kleisin subunit and two ATP molecules [6,11,12]. These interactions at both the head and Hinge domains give Smc dimers the potential to form large rings that have been observed in preparations of purified Smc complexes [13].

One of the most studied Smc complexes is cohesin, which mediates sister chromatid cohesion. Cohesin is composed of Smc1, Smc3, Scc3, and the kleisin subunit Mcd1/Scc1 [14–18]. The association of cohesin molecules with chromatin requires the integrity of all cohesin subunits and the ability of the Smc molecules to bind and hydrolyze ATP [19–22]. Multiple cohesins bind proximal to each centromere, forming a large pericentric domain. Cohesins also bind along chromosome arms. In budding yeast, these cohesin-associated regions (CARs) extend over approximately 1 kb of DNA and are spaced at roughly 10-kb intervals [23–25].

Several auxiliary factors contribute to the establishment, maintenance, and eventual dissolution of sister chromatid cohesion. The binding of cohesin to chromosomes at any phase of the cell cycle requires the loading factors Scc2 and Scc4 [26]. However, only cohesin binding in S phase, coupled with the function of Ctf7, results in the establishment of cohesion [16,27]. Pds5 binds cohesin and helps maintain cohesion during S and G2 phases of the cell cycle [28–30]. Finally, separase promotes removal of cohesin at the onset of anaphase by cleavage of Mcd1 [31].

These observations have led to two distinct models to explain how cohesin molecules crosslink sister chromatids. The embrace model posits that cohesin rings encircle chromosomes prior to replication and make no specific contacts with chromatin [6,20]. A topological interaction between cohesin and chromatin is supported by the fact that cohesin can be released from chromatin either by a single cleavage of the DNA or a single proteolytic cleavage of a cohesin subunit [19,20,32]. Passage of the replication fork through cohesin rings leaves both sister chromatids trapped inside, establishing sister chromatid cohesion. In contrast, oligomerization models, on the basis of observations of other Smc complexes, posit that cohesins bind to both sister chromatids. Then, cohesins on one sister chromatid oligomerize with cohesins on the other sister chromatid to generate cohesion [33–36]. To resolve these and other models will require a better understanding of how cohesins bind chromatin and the relationship between this chromatin binding and establishment of cohesion.

Mutagenesis of cohesin subunits provides one approach to gain insight into the chromatin binding of cohesin mutants. Indeed, site-directed mutagenesis of conserved Smc1 residues in the Hinge, Walker A, Walker B, and Signature motifs demonstrated that Smc1 must bind Mcd1 and Smc3, as well as bind and hydrolyze ATP in order for cohesin to bind chromatin [7,21,22,37]. While informative, these mutational analyses of cohesin–chromatin association leave key questions unanswered. Do these mutations define all the domains of cohesin subunits required for chromatin binding? Is chromatin binding of cohesin anywhere on chromatin prior to DNA replication sufficient to generate cohesion? Are additional constraints on cohesin necessary to generate cohesion?

To address these types of questions, one needs a way to identify rare mutant forms of cohesin that modulate rather than eliminate its activity. In the past, knowledge of a protein's structure (sites for modification or interaction with other subunits) has been used to make dominant negative mutants that alter its activity or incorporation into a fully functional complex. We rationalized that the reciprocal would also be true; surveying an entire polypeptide chain for rare insertions with a dominant negative phenotype should provide a highly efficient means to identify precise regions of a protein important for its activity and/or assembly with other subunits. Furthermore, the study of these alleles should be highly informative for elucidating the molecular mechanism of a protein/complex. With this in mind, we screened a library of random insertion mutations in *SMC1* for those that cause a dominant negative phenotype in the budding yeast, Saccharomyces cerevisiae. This strategy is henceforth referred to as random insertion dominant negatives (RID). Here, we successfully use RID to identify rare *SMC1* alleles that are highly informative in dissecting Smc1′s role in the assembly and function of cohesin. We also use these mutants along with a *ctf7* mutant to provide important insights into cohesin and sister chromatid cohesion.

## Results

### RID Screen Defines Five Regions Necessary for Smc1 Activity and the Establishment of Cohesion

As the basis of our RID mutagenesis of *SMC1,* we constructed a minichromosome containing the *SMC1-TAP* gene under control of the galactose-inducible *GAL1* promoter (see Materials and Methods). The level of Smc1-TAP protein expressed from the *GAL1* promoter relative to the endogenous *SMC1* promoter decreases 0.5-fold under uninducing conditions and increases 100-fold under inducing conditions (unpublished data). The Smc1-TAP protein is functional, as it restores viability and normal growth rate to cells carrying the temperature-sensitive *smc1–2* allele strain deleted for the essential *SMC1* gene under repressing, uninducing, and inducing conditions (Figure S1 and unpublished data). The *SMC1-TAP* gene was mutagenized by a Tn7-based in vitro system that resulted in five amino acid insertions at random positions within the Smc1 protein. For brevity, the initial *SMC1-TAP* gene is henceforth referred to as the *SMC1* or wild-type allele, and the insertion derivatives are named based upon the position of the insertion in the amino acid sequence.

Minichromosomes harboring the mutagenized library of *SMC1* were transformed into budding yeast and assayed for their effects on cell viability and cohesion. A total of 13 transformants out of 2,500 candidates showed a marked decrease in cell viability under inducing conditions (Figure S1). The fact that these transformants are viable under uninducing conditions, where significant mutant smc1 expression occurs, indicates that these mutants are not dominant under low expression. To examine whether the mutant proteins are functional, minichromosomes containing inducible wild-type *SMC1* or dominant negative *smc1* alleles were introduced into cells carrying the temperature-sensitive *smc1–2* allele at the endogenous locus [17]. Under uninducing or inducing conditions, the temperature sensitive growth of the *smc1–2* strain is complemented by expression of wild-type *SMC1*, but not the insertion alleles (Figure S1B), indicating that the products of the insertion alleles are defective for Smc1 function under all conditions. The *smc1–2* strains were then used to test the ability of the *smc1* insertion alleles to generate cohesion. All *smc1* insertion alleles are dramatically impaired for the establishment and maintenance of sister chromatid cohesion (unpublished data) (Figure 1B–1D).

**Figure 2:**
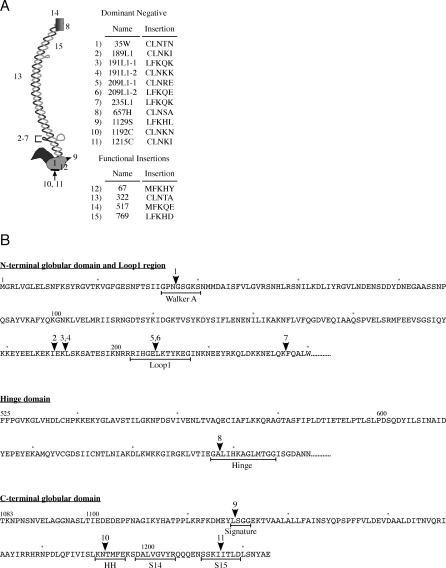
Insertion Mutations Disrupt Smc1 Function in Sister Chromatid Cohesion (A) A screen for dominant negative *smc1* alleles identified 11 unique insertion mutations that cluster to previously characterized motifs in Smc1 (1, 8, 9) and uncharacterized Loop1 region (2–7, 10, 11) of Smc1. Clustering of insertions in these regions was a consequence of the dominant negative selection as evidenced by the different position of four functional insertion alleles (12–15) that can that rescue growth of an *smc1–2* strain. (B) Position of the insertions and the relevant motifs in Smc1. Insertion names are based upon the position of the insertion in the primary amino acid sequence and the motif affected C, C terminus; H, Hinge; L1, Loop1 region; S, Signature motif; W, Walker A.

All 13 insertions mapped to the *SMC1* ORF, and 11 of them were unique (Figure 2A and 2B). A total of five insertions map to regions of known functional importance: the Walker A motif (35W), Signature motif (1129S), globular Hinge region (657H), and C terminus of Smc1 (1192C and 1215C) [7,21,22]. The remaining six insertions are either in 209L1–1 and 209L1–2 or cluster around Loop1 (189L1, 191L1–1, 191L1–2, and 235L1). Loop1 was previously defined through bioinformatics as one of three small regions within the α-helical domains of Smc molecules that are predicted to disrupt coiled-coil formation [38]. Since the insertions within and around Loop1 exhibit similar phenotypes (see below), these insertions define a new functional region of Smc1, which we call the Loop1 region.

### A Subset of RID Mutants Disrupt Cohesin Complex Assembly

**Figure 3:**
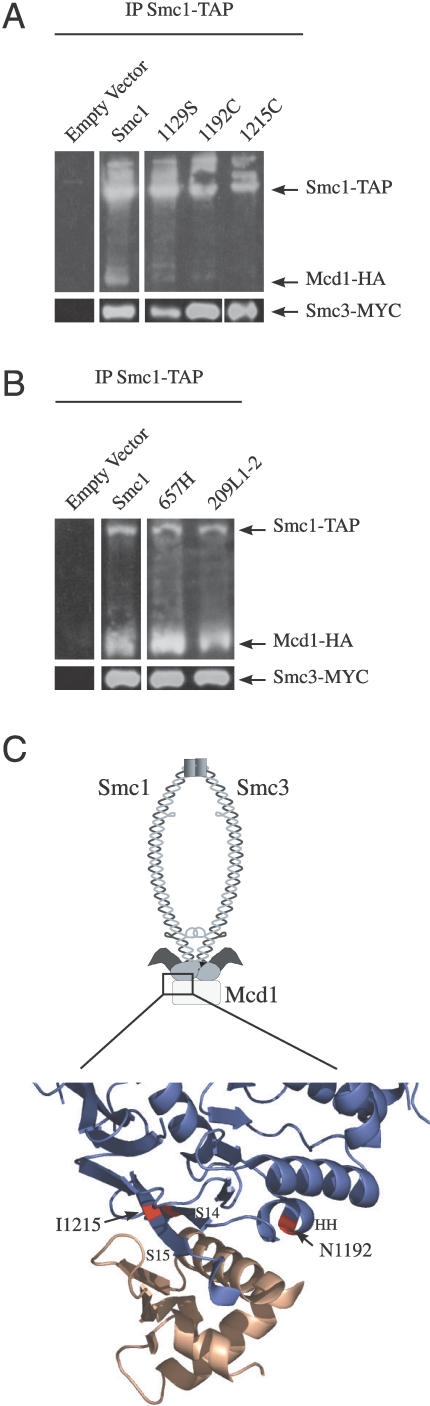
A Subset of *SMC1* RID Alleles Disrupt Cohesin Complex Assembly Protein extracts were prepared from the strain 1377 A1-4B *(MCD1-6HA)* or 2258G *(SMC3-12MYC)* containing minichromosomes with the indicated *SMC1* alleles. Cells were grown under repressing conditions to assure physiological levels of the different Smc1 proteins. Smc1 proteins were immunoprecipitated and separated by SDS-PAGE. Immunoblots were probed to detect Smc1-TAP, Mcd1-6HA, and Smc3-12MYC (see Materials and Methods). (A and B) None of the *Smc1* RID alleles disrupt the physical association with Smc3-12MYC. (A) Alleles in the Signature motif, HH Helix, S15 β-strand, and Walker A motif (unpublished data) of Smc1 disrupt the physical association of Mcd1-6HA, whereas (B) alleles in the Hinge and Loop1 regions do not. (C) A region of the cocrystal of Smc1 (blue) and Mcd1 (brown) is shown (PDB 1W1W). Helix HH and the β-sheets S14 and S15 are indicated. The amino acid positions of 1215 and 1192 are indicated (red).

We wanted to determine the efficacy of RID mutagenesis to define regions of Smc1 important for cohesin assembly. For this purpose we analyzed the ability of the 11 RID smc1 mutants (expressed at physiological levels) to coimmunoprecipitate with Mcd1 and Smc3. By comparison with wild-type Smc1, four RID mutants are dramatically reduced in their ability to coimmunoprecipitate with Mcd1, while all can coimmunoprecipitate Smc3 (Figure 3A and 3B). The failure to identify insertions that block Smc3 association is not surprising, since mutants defective in Smc1/Smc3 dimerization do not interact with any other cohesin subunit [21,22,39] and, therefore, are not likely be dominant negative.

The four Smc1 insertions defective for Mcd1 binding lie within the Walker A motif (35W), the Signature motif (1129S), the HH helix (1192C), or the S15 β strand (1215C) (Figures 2B and 3C). These are four of the five motifs known to be required for Mcd1 binding. The Walker A and Signature motifs, through ATP binding, tether together the Smc1 and Smc3 head domains so that they can both associate with a single Mcd1 [21,22,39]. The helix HH and S15 β strand provide two of the three major contacts between Smc1 and Mcd1 (Figure 3C) [39]. These motifs are widely spaced within the polypeptide chain and constitute a target of only ∼2% of the total residues. The efficient identification of these small disperse motifs by RID validates it as an extremely efficient means to identify regions of proteins important for complex assembly.

### Smc1, Loop1, and Hinge Alleles Assemble into a Cohesin Complex that Binds Chromosomes

**Figure 4:**
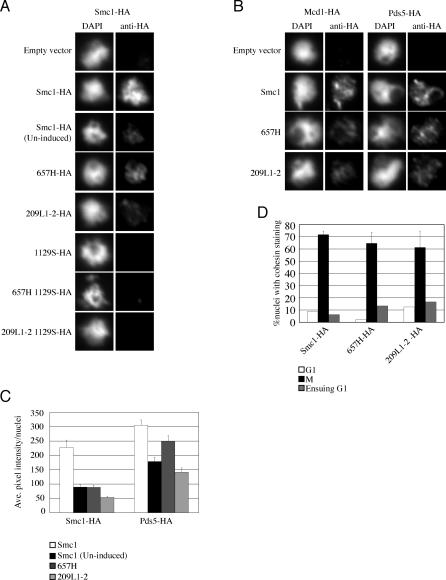
*Smc1* RID Mutants *657H* and *209L1–2* Bind Chromosomes as Subunits of Cohesion (A and B) Minichromosomes with the different *Smc1* alleles were introduced into YMM10 *(smc1–2),* YMM11 *(smc1–2 MCD1-6HA),* or YMM20 *(smc1–2 PDS5-6HA).* Cultures of these cells were released from a G1 arrest into media that supported either the induced or uninduced expression of the *Smc1* alleles, inactivated smc1–2, and caused arrest in mitosis (see Materials and Methods). Nuclei from mitotic arrested cultures were spread on slides and processed for immunofluorescence. The micrographs show representatives images of chromosomes (DAPI) and the chromosome staining of the different Smc1 proteins, Mcd1-6HA, and Pds5-6HA (anti-HA). The allele of *SMC1* expressed from the minichromosome is listed on the left. (C) The chromosome association of Smc1-HA and Pds5-6HA was quantitated by determining the average pixel intensity per nuclei following staining for Smc1-HA and Pds5-6HA (see Materials and Methods). (D) Nuclear spreads were prepared as described above, except half of the cells released from G1 were arrested in mitosis while the other half were arrested in the ensuing G1 (see Materials and Methods). Nuclear spreads were prepared from aliquots of the original G1-arrested cells and those arrested in mitosis and the ensuing G1. The percent of nuclei with chromosomal staining of Smc1-HA, 657-HA, and 209L1–2-HA was determined.

In addition to being a powerful tool to dissect cohesin assembly, we anticipated that RID would also efficiently identify informative alleles for cohesin function. The remaining seven RID alleles in the Hinge and Loop1 regions encode mutant Smc1 proteins that assemble with Smc3 and Mcd1 (Figure 3B) as well as Scc3 (unpublished data). Since they assembled with all known cohesin subunits, they were candidates for alleles that blocked cohesin function. All previously published alleles of cohesin subunits block chromatin binding. Therefore, we tested whether cohesins with these RID smc1 mutants were competent for chromosome binding. Minichromosomes were generated that express wild-type Smc1, 657H, or 209L1–2 fused to a 3XHA epitope, again under control of the *GAL1* promoter. These alleles will henceforth be referred to as *Smc1-HA, 657H-HA,* and *209L1–2-HA,* respectively. Cultures of *smc1–2* cells containing these minichromosomes were released from G1 under conditions that inactivate the smc1–2 protein and induce expression of the galactose-regulated allele. These cells were then arrested prior to anaphase. Nuclei from these cells were spread on slides and processed for immunofluorescence to detect the chromosome association of different Smc1 proteins (Figure 4A). Like wild-type *Smc1-HA,* the insertion alleles *657H-HA* and *209L1–2-HA* associate with chromosomes.

We also monitored the chromosome localization of epitope-tagged Mcd1, a cohesin subunit, and Pds5, a cohesin accessory factor whose chromatin binding is mediated by cohesin (Figure 4B) [29,30]. Epitope-tagged Mcd1 and Pds5 localize to chromosomes in cells expressing the wild-type 657H or 209L1–2 allele, but not in cells with only inactivated *smc1–2* (empty vector). The fact that the 657H and 209L1–2 alleles mediate Mcd1 and Pds5 localization to chromosomes suggests that these smc1 alleles bind chromosomes as part of the cohesin complex.

While it is clear that upon induction, the Hinge and Loop1 mutant cohesins can associate with chromosomes, their levels of chromosomal staining are reduced compared to induced wild-type cohesin (Figure 4C). Thus, the first question we wanted to ask was whether this reduction was sufficient to explain the cohesion defect of the Hinge and Loop1 mutants. To assess the level of cohesin binding to chromosomes, cells expressing Smc1, 657H, or 209L1–2 were arrested in mitosis. Nuclear spreads were prepared, and the levels of Smc1 and Pds5 immunostaining on chromosomes were quantified (Materials and Methods). The levels of chromosome binding for induced 657H-HA and its associated Pds5 are nearly identical to the binding of Smc1-HA and Pds5 under uninducing conditions (Figure 4C), a level of binding that is sufficient to generate cohesion and normal cell growth. Therefore, the level of chromosome association for 657H complexes should be sufficient to generate cohesion. The levels of 209L1–2-HA binding and its associated Pds5 are reduced by a maximum of 40%. This reduction may be below a threshold needed to generate cohesion. However, 50% of chromosome-bound cohesins in yeast meiosis and 90% in mammalian mitosis can be removed without eliminating cohesion [40,41]. Therefore, the level of chromosome association for 209L1–2-HA complexes is also likely to be sufficient to mediate at least partial sister chromatid cohesion, yet this seems not to be the case (Figure 1C).

If the quantity of chromatin binding for the mutant complexes is sufficient to generate cohesion, then the quality of their chromatin binding must be defective. The mutant complexes may bind chromatin by a nonphysiological mechanism. Previous studies have shown that the binding of cohesin to chromatin requires Mcd1 and ATP. The ATP dependence is mediated through the Walker A, Walker B, and Signature motif. To test whether the mutant complexes also bound in an ATP-dependent manner, we constructed Hinge and Loop1 mutants that carried a second insertion allele in the Signature motif. While the protein products of these double mutants are stable (unpublished data), they failed to associate with the chromatin (Figure 4A), indicating that the mutant complexes bind by ATP-dependent mechanism. One method to address whether the mutant cohesin complexes require Mcd1 to bind chromosomes would be to inactivate the temperature sensitive mcd1–1 in strains harboring the smc1–1 and the smc1 loop or hinge mutants. Unfortunately, mcd1–1 and smc1–1 are synthetically lethal. As an alternative, we followed the chromosome association of the Hinge and Loop1 mutant complexes through a cell cycle. Cohesin binds chromosomes only after G1, when Mcd1 is expressed [14,15], and dissociates from chromosomes upon the onset of anaphase, when Mcd1 is cleaved [19]. Cohesin with 657H-HA or 209L1–2-HA is absent from chromosomes in G1, localizes to chromosomes in metaphase, and is lost by the following G1, a pattern identical to cohesin with Smc1-HA (Figure 4D), strongly suggesting that the mutant complexes, like wild-type complexes, require Mcd1. The fact that, like wild- type, the Hinge and Loop1 mutants bind chromatin in an ATP- and Mcd1-dependent manner suggests that they all bind chromatin by a similar mechanism.

**Figure 5:**
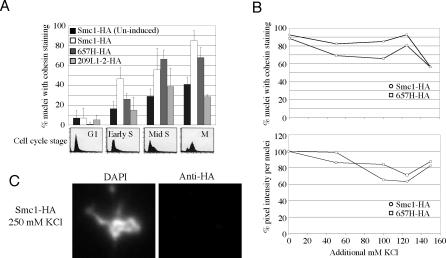
Binding of Cohesins to Chromosomes in S Phase Is Not Sufficient to Establish Cohesion (A) MM10 cells containing minichromosomes with different *Smc1* alleles were released from G1 in media that supported either induced or uninduced expression of the mutant *Smc1* alleles, inactivated smc1–2, and prevented progression beyond metaphase (see Materials and Methods). Aliquots of cells were removed at specific time intervals after release from G1. These aliquots were processed for flow cytometry to determine DNA content and used to prepare spread nuclei to follow chromosome staining of the different Smc1 proteins. The percent of nuclei with chromosomal staining was determined as well as the average pixel intensity per nuclei (unpublished data). (B) To assess the stability of this chromosome-associated cohesin, mitotic-arrested nuclei were spread on slides in the absence of fixative and incubated in buffer containing different amounts of additional KCl. The percent of nuclei with chromosomal staining and the average pixel intensity per nuclei were determined. The average pixel intensity is expressed as a percentage of the intensity in the absence of additional salt. (C) A micrograph of a representative nucleus (DAPI) and the chromosome staining of the Smc1-HA (anti-HA) after treatment with 250 mM KCl.

Despite these similarities to wild-type chromatin binding, the mutant complexes could still fail to generate cohesion if their binding is too late in the S phase to establish cohesion or too unstable to maintain cohesion until M. To test if the mutant cohesins bind chromatin in a timely manner, cells were released synchronously from a G1 arrest and analyzed at different intervals for DNA content and chromosome association of wild-type and mutant cohesins (Figure 5A). The timing of chromosome association during S phase for 657H-HA or 209L1–2-HA cohesins is very similar to that seen for uninduced levels of Smc1-HA cohesin, which is sufficient to generate cohesion. Also, the amount of cohesin bound per nuclei during S phase increased with similar kinetics for Smc1-HA, 657H-HA, and 209L1–2-HA cohesins (unpublished data). Hence, cohesin containing 657H or 209L1–2 exhibits a normal timing of chromosome association.

To examine the stability of chromosome binding for cohesin, nuclei were prepared from mitotic cells expressing Smc1-HA, 657H-HA, or 209L1–2-HA and spread in the absence of fixative. Spread nuclei were incubated in ∼50 ml buffer with varying amounts of KCl for 30 min. After incubation, fixative was added, and the association of cohesin with chromosomes was examined by immunofluorescence (Figure 5B). No change in chromosome binding is observed for Smc1-HA in the presence of 150 mM KCl, corroborating previous biochemical analyses that infer stable association between cohesin and chromosomes [32]. Similarly, no significant change in chromosome binding is observed for 657H-HA. Both wild-type and mutant cohesins are extracted completely from spread chromosomes at 250 mM KCl (Figure 5C, unpublished data). Similar results were obtained with the 209L1–2-HA mutant (unpublished data). Therefore, cohesin complexes with wild-type, 657H-HA, or 209L1–2-HA appear to exhibit the same stability of chromosome binding under these conditions. Together, our analyses of the Hinge and Loop1 mutants reveal a new type of cohesin complex. Like wild-type, these mutant complexes bind stably to chromatin, bind chromatin with proper cell cycle timing, and require ATP and Mcd1. However unlike wild-type, these mutant complexes fail to generate cohesion. These mutants also suggest that binding, per se, prior to DNA replication is not sufficient to generate cohesion.

### Smc1, Loop1, and Hinge Regions Are Required for Cohesin Enrichment at CARs

**Figure 6:**
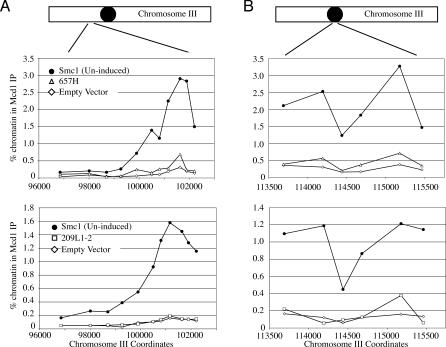
657H and 209L1–2 Disrupt CAR Enrichment of Cohesion Cultures of YMM11 *(smc1–2 MCD1-HA)* cells containing minichromosomes with different *Smc1* alleles were grown and arrested in metaphase (Figure 4C, see legend). Cells were processed for ChIP (see Materials and Methods). Mcd1-HA binding at specific chromosomal loci was analyzed by PCR. Data shown is for (A) the *CARC1* locus, located 11 kb from the centromere of Chromosome III, and (B) 2 kb surrounding *CENIII*.

Since we could not explain the cohesion defect of these mutants by changes in their general chromosome-binding properties, we tested the specificity of their chromatin binding by examining their enrichment at CARs using chromatin immunoprecipitation (ChIP) [23–25]. Cohesin enrichment was analyzed initially at the centromere of Chromosome III and *CARC1,* a CAR approximately 11 kb from the centromere (Figure 6A and 6B). Under uninduced levels of Smc1 expression, Mcd1-6HA is enriched at *CARC1* and around *CEN3* as reported previously [24,25]. Conversely, Mcd1-6HA enrichment at *CARC1* and *CEN3* is eliminated for cohesin complexes containing 657H or 209L1–2. Similar results were obtained for *CARL1* on Chromosome XII (unpublished data). Therefore, cohesin complexes with 657H or 209L1–2 fail to be enriched at CARs.

Because these mutant complexes do bind to chromatin as assayed by chromosome spreads, they apparently are bound to sites other than CARs. One possibility is that the mutant cohesins bind to chromatin through the Scc2/Scc4 loading factor, but are trapped in a nonproductive preloading complex. To test this, we examined cohesin binding at Scc2/Scc4 chromatin-binding sites [42], but we observed no enrichment of the mutant cohesins at these sites (Figure S2). The failure to observe enrichment of the mutant cohesins in our ChIP experiments does not exclude the possibility that they bind randomly within these regions. The enrichment of wild-type cohesin at CARs is only 10-fold above background, and CARs are spread at approximately every 10 kb, hence the dispersal of this signal to random sites would dilute the signal to background levels. While the position of the ectopic binding remains to be elucidated, the fact that the Hinge and Loop1 mutants cause ectopic binding indicates that Smc1 plays an active role in the specific localization of cohesins to CARs and pericentric regions. Furthermore, since this mislocalization is the only severe cohesin defect we have been able to observe in the Hinge and Loop1 mutants, it suggests that cohesin localization to CARs is critical for cohesion.

### CAR Enrichment Is Not Sufficient for Cohesin Function

Our studies show that the Hinge and Loop1 regions of Smc1 are required for the establishment of cohesion and the enrichment of cohesin at CARs and pericentric regions. The accessory protein Ctf7/Eco1 is also required for the establishment of cohesion [16,27]. In a *ctf7* mutant, cohesins pellet with chromatin [16]. This technique does not distinguish between ectopic and specific chromatin binding. Indeed, if *ctf7*, like Hinge and Loop1 mutants, showed ectopic binding, then this would suggest that Ctf7 interacts with the Hinge and Loop1 regions of Smc1 to ensure specific binding to CARs.

**Figure 7:**
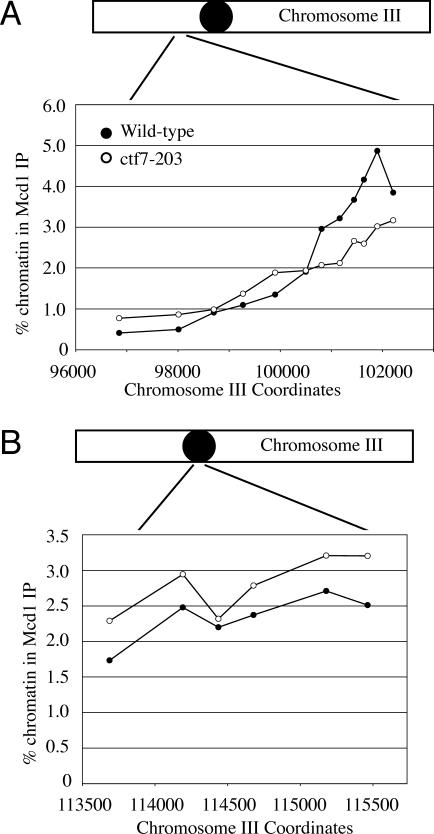
Ctf7 Functions after Cohesin Enrichment at CAR Sites and Pericentric Regions Wild-type *CTF7* and temperature-sensitive *ctf7–203* mutant cells containing Mcd1-HA were released from G1 in media at 37 °C with HU to inactivate ctf7 and to arrest cells in early S phase. Cells were processed for ChIP to assess cohesin binding at (A) *CARC1* locus and (B) *CENIII*.

To test the function of Ctf7 in cohesin enrichment at CARs, we compared the localization of cohesin on chromatin in wild-type and *ctf7* temperature-sensitive strains. Wild-type and *ctf7* mutant cells expressing Mcd1-HA as the sole source of Mcd1 function were synchronized at the permissive temperature of 23 °C in G1. These cells were released to the the nonpermissive temperature of 37 °C in media containing hydroxyurea (HU) to inactivate mutant ctf7 protein and block DNA replication [27], respectively. Cohesin association to chromatin was monitored by ChIP (Figure 7A and 7B). In wild-type cells arrested in HU, cohesin is enriched at CARs and pericentric regions as described previously [24,25]. In *ctf7* strains arrested in HU at the nonpermissive temperature, cohesin is again enriched at CARs and pericentric regions. Thus Ctf7 is not needed to localize cohesins to CARs or pericentric regions.

The above results indicate that Ctf7 function is distinct from that of the Hinge and the Loop1 regions of Smc1. Previous experiments showed that Ctf7 function is required during S phase; however, this activity was not ordered relative to cohesin loading at CAR sites. To test whether Ctf7 is required coincident with or after cohesin binding to CARs, wild-type and *ctf7* mutant cells were allowed to progress from G1 to early S (HU arrest) at the nonpermissive temperature and then shifted to the permissive temperature for the remainder of the cell cycle. The results show that cell viability was 93% ± 4% for wild-type cells and 88.2% ± 5% for *ctf7* mutant cells, indicating that Ctf7 function is required after cohesin loads to CARs. This result is strongly supported by cell cycle mapping studies that show inactivation of *ctf7* from early S (HU arrest) and prior to the end of DNA replication leads to cell death [16,27,43], indicating that Ctf7 performs its essential function during this window of the cell cycle. Thus, Ctf7 appears to function in the establishment of cohesion after the Smc1-, Loop1-, and Hinge-dependent localization of cohesin to CARs and pericentric regions, and during S phase.

## Discussion

Our results validate RID as a very efficient strategy to identify Smc1 alleles that provide important structural and functional information about Smc1 and cohesin. First, of the 11 smc1 RID alleles, ∼40% blocked Smc1 association with Mcd1 but not Smc3. Thus, RID efficiently identifies alleles that trap partially assembled cohesin. Furthermore, these Smc1 insertions lie within four of the five small structural elements (each element is approximately ten residues each spread throughout a total of 1,300), which are required for Mcd1 binding. The efficacy and remarkable precision of RID suggests that it can be used effectively on less well-characterized proteins for the de novo identification of candidate regions of that protein that mediate its binding to interacting partners. Second, the remaining seven RID alleles of Smc1 identify a new functional domain (the Loop1 region), and a new function for the Hinge beyond its established function in dimerization. These Hinge and Loop1 alleles also generated cohesin complexes that unlike any previous cohesin mutants retain the ability to bind chromatin. The isolation of these unusual alleles underscores the combined power of using random mutagenesis, which allows one to avoid the inherent biases of directed mutagenesis, and the imposition of the dominant negative phenotype, which allows rapid identification of rare partially functional alleles in a sea of common null mutations caused by misfolding and truncations. Given the success of the RID strategy for Smc1/cohesin, RID should be a useful tool to dissect the structure and function of many multi-subunit complexes.

Our study of RID smc1 alleles has provided important new insights into cohesin binding to chromatin. First, our results show that in vivo the Hinge and Loop1 regions are needed for binding to CARs, two domains not implicated in chromatin binding by either the embrace or snap model. The joint requirement for these two domains is even more surprising given their apparent physical separation (∼40 nm) based upon electron micrographs of cohesin [13]. Interestingly, in vitro analyses of bacterial Smc complexes have implicated the hinge domain in single-stranded and double-stranded DNA binding [37,44] and DNA-dependent stimulation of the ATPase activity of the head [44]. Thus, our in vivo study combined with these in vitro studies support DNA-dependent functional interactions between the opposite ends of Smc complexes.

The second interesting feature of cohesins containing the RID smc1 mutants is that they appear to bind chromatin ectopically. This conclusion is based upon the observations that cohesins exhibit general chromatin binding as assayed by chromatin spreads but are no longer enriched at CARs. It is possible that the cohesin binding is compromised at CARs in some way that they bind there but subsequently dissociate. Consistent with this, the level of binding of overexpressed RID mutant proteins is reduced compared to overexpressed wild-type protein. However, under our assay conditions using chromatin spreads, the Hinge, Loop1, and wild-type cohesins appear to be bound with similar stability. Furthermore, the level of chromatin binding of cohesin, when the Hinge mutant is overexpressed, is still comparable to the level of binding for uninduced wild-type, which is sufficient for cohesion function. Alternatively one could argue that the ectopic binding observed by chromatin spreads reflects some artifact of this assay. This is extremely unlikely, as chromatin binding observed by spreads for the RID mutants share many of the chromatin-binding features of wild-type cohesin, including Mcd1 dependence, ATP dependence, and proper cell cycle loading in S and unloading in M. Together these results suggest that cohesins are capable of general chromatin binding, mediated by Mcd1 binding and ATP functions, but are then targeted to CARs through an additional function(s) mediated by the Hinge and Loop1 regions.

In one scenario the Loop1 and Hinge regions may target cohesins to CARs by binding targeting factors that recognize specific chromatin features; the RID mutants perturb the binding of the targeting factors. There are precedents for this idea, since the Hinge and Loop1 regions mediate interactions with non-SMC factors in other Smc-related proteins [45–47]. In addition, histone modifications have already been demonstrated to be critical to target cohesin binding to heterochromatin and double strand breaks [48–50]. In this model, cohesin binding to chromatin resembles RNA polymerase; both are targeted to specific sites through specificity factors that recognize changes in chromatin, and in their absence load with lower efficiency at abundant cryptic sites.

As an alternative, cohesin itself may be capable of recognizing specific features of chromatin. Indeed the MutS mismatch repair complex, which shares similarities to Smc complexes, forms a ring that topologically traps generic DNA, while residues within the ring specifically recognize mismatch DNA [51–53]. In this scenario, the Smc1, Hinge, and Loop1 mutant subunits allow a topological interaction of cohesin with chromatin, but perturb its ability to make intimate chromatin/DNA interactions. Interestingly, in vitro studies have implicated residues on the interior of the bacterial Smc ring for DNA binding and DNA-dependent stimulation of the ATPase [44].

The chromatin-binding properties of cohesin in *ctf7* and RID mutants also provide important insights into the mechanism of sister chromatid cohesion. First, the binding of cohesin to chromatin in early S at CARs (*ctf7* mutants) or ectopic sites (Hinge and Loop1 mutants) is not sufficient to generate cohesion. Second, cohesin binding to CARs and centromeres appears necessary for cohesion. Third, an auxiliary factor, Ctf7 enables cohesins already bound at CARs to generate functional cohesion. These observations all contradict a one-step mechanism for cohesion like the simple embrace model, which requires only chromatin binding of cohesin anywhere on the chromosomes followed by DNA replication.

Rather, our results support tethering of sister chromatids by two chromatin-binding events, which require specific binding of cohesins at CARs followed by a Ctf7-dependent step. Given these new constraints, we propose the following working model for the establishment of cohesion: cohesins bind to CAR sites as they emerge from the replication fork and are subsequently activated by Ctf7 to initiate the capture of the homologous CAR on the sister chromatid. This capture could occur by activating a second chromatid binding event by a single complex (for example, a second embrace) or by activating oligomerization of cohesins bound to each CAR (the oligomerization models) [33–36]. In either version of this two capture model, the specific binding to CARs and Ctf7 steps would be critical. Because of local proximity during replication, a cohesin molecule bound to a CAR on one sister chromatid will find the sister CAR and/or cohesin bound to that site before finding other sites/cohesins in the genome. We speculate further that the activation by Ctf7 is programmed to be local/transient. As a result, cohesin bound to a random site on a chromatid will be unlikely to remain active long enough to find a random CAR or another randomly bound cohesin. Thus, the targeting of cohesin by CAR binding and its local activation by Ctf7 would provide spatial and temporal regulation of cohesin to activate the second capture and ensure that cohesin generates a crosslink only between only sister chromatids and not random chromatin. Finally, the specificity of tethering by Smc complexes in other DNA processes may be achieved through specific chromatin binding coupled with Ctf7-like activators.

## Materials and Methods

### Media and strains.

Yeast strains were grown in YEP, SC-URA, or SC-URA-TRP media [54] supplemented with 2% dextrose (D), 2% raffinose 2% galactose (RG), 3% glycerol 2% lactic acid (LA), or 3% glycerol 2% lactic acid 2% galactose (LAG), as indicated. Glucose, raffinose, and galactose were purchased from Sigma-Aldrich (http://www.sigmaaldrich.com), glycerol from EMD Biosciences (http://www.emdbiosciences.com), and lactic acid (40% v/v stock, [pH 5.7]) from Fisher Scientific (http://www.fishersci.com). A PCR-based strategy was used to generate a complete genomic replacement of *SMC1* with the *Schizosaccharomyces pombe His5+* gene [55], creating the yeast strain Ymm1. Ymm1 is dependent on the plasmid pMM26 for viability. The genotypes of strains used in this work can be found in Table S1. Yeast transformation and genetic methods were as described previously [56].

### Plasmids.

The plasmids described were generated through use of the Echo Cloning System (Invitrogen, http://www.invitrogen.com), which results in the expression of genes fused to COOH-terminal V5 epitopes under control of the *GAL1* promoter. In all cases, the V5 epitope was replaced by a TAP tag using a PCR-based tagging strategy [57,58]. The plasmid pMM14 contains the *SMC1* ORF. Restriction digestion by SmaI and PmeI, followed by religation, destroyed a unique PmeI site and generated AMH4.

The plasmid pMM26 was derived from pMM14. *GAL1* was replaced with a 412-basepair fragment immediately 5′ of the *SMC1* ORF using AgeI and XhoI restriction sites, creating pMM24. Loss of the *URA3* gene by BsgI digestion, followed by T4 blunting and religation, generated pMM26. Expression of Smc1-TAP from pMM24 and pMM26 is capable of rescuing growth of a temperature sensitive *smc1–2* strain (1360–7C) and a strain deleted for endogenous *SMC1*. The plasmid pMM25-3HA expresses Smc1 fused to a COOH-terminal TAP-3HA tag under control of the *GAL1* promoter. Through a PCR-based strategy, a BamHI site within the TAP coding sequence of pMM14 was destroyed by a silent mutation and replaced by a BamHI site immediately 5′ of the TAP stop codon [57]. *TRP1* was lost by SmaI digestion, followed by religation, resulting in pMM14-PIBS. The XbaI/SmaI fragment of pMM14-PIBS was subcloned into pRS305, resulting in pMM27. A 3XHA coding sequence was inserted in frame at the BamHI site of pMM27, resulting in pMM27-3HA. Replacing the XbaI/SmaI fragment of pMM25 with the XbaI/SmaI fragment of pMM27-3HA resulted in pMM25-3HA. The plasmid pMM25-3HA is able to rescue viability of an SMC1 deleted strain under either uninduced or induced expression conditions. The plasmids 209L1–2-HA and 657H-HA were generated by replacing the XhoI/XbaI fragment of pMM25-3HA with those from 209L1–2 and 657H.

### Construction of a mutant smc1 library.

Insertion mutagenesis of AMH4 was performed using the GPS-Linker Scanning system (New England Biolabs, http://www.neb.com). In vitro mutagenized AMH4 was transformed into Escherichia coli. Approximately 3,100 colonies were scraped from plates, and plasmid DNA was isolated using a Plasmid Maxi kit (Qiagen, http://www.qiagen.com). The majority of plasmids received only a single insertion (unpublished data). Plasmid DNA in the primary library was linearized by PmeI digestion, gel purified, religated, and transformed into E. coli. Plasmid DNA from this secondary library was isolated from approximately 4,000 colonies from plates using the Plasmid Maxi kit.

### Screen for dominant negative and functional *smc1* alleles.

The secondary library was transformed into YMM1/pMM26, with transformants selected for on SC-URA-TRP-D plates at 23 °C. Individual transformants were picked and patched to new SC-URA-TRP-D plates and grown at 23 °C. Patches were replica plated to an identical plate and a SC-URA-TRP-RG plate and grown for 3–5 d at 23 °C. From patches that were impaired for growth on SC-URA-TRP-RG plates, plasmids were isolated from the identical patch growing on the SC-URA-TRP-D plate and retested. For plasmids that retested, the insertion mutations were mapped by restriction endonuclease digestion and sequenced. To isolate functional alleles, the secondary library was transformed into an *smc1–2* strain, 1360–7C. Transformants were selected for on SC-URA-D plates at 23 °C, patched to new SC-URA-D plates, and grown at 23 °C. Patches were replica plated to an SC-URA-RG plate and grown at 37 °C. Plasmids were isolated from patches that grew at 37 °C and retested. For plasmids that retested, the insertion mutations were mapped by restriction endonuclease digestion and sequenced.

### Cell synchronization.

Exponentially dividing cell cultures were initially grown in SC-URA-LA at 23 °C. α-Factor (1.5 × 10^−8^ M [Sigma]) was added to cultures in mid-log phase (approximately 0.5 × 10^7^ cell/ml). To simultaneously inactivate *smc1–2* and release them from G1 arrest, cells were washed twice in either 37 °C YEP-LA or 37 °C YEP-LAG containing 0.1 mg/ml Pronase (Sigma). Cells were resuspended in either 37 °C YEP-LA or YEP-LAG and grown at 37 °C. To arrest G1 released cells in metaphase, nocodazole was added to a final 15 μg/ml (1.5 mg/ml in DMSO stock [Sigma]) and cultures were grown for 3 h. To arrest in early S phase, G1 released cells were grown in the presence of 0.2 M hydroxyurea (HU) (Sigma) for 3 h. Cell cycle arrest was assessed by flow cytometry and cell morphology [27].

### GFP assay for sister chromatid cohesion, flow cytometry, immunoblotting, coimmunoprecipitation, and ChIP.

Cells were processed to visualize GFP by microscopy or to measure DNA content by flow cytometry as described previously [27,59]. Protein extracts for coimmunoprecipitation and immunoblotting were prepared as described previously [15,20] from cultures grown in YEP-D media. ChIP was performed as described [60]. Information about the primers used in this study is available upon request. PCR and data analysis for ChIP was performed as described [49]. All experiments were done at least twice and a representative dataset is shown.

### Chromosome spreads and immunofluorescence.

Chromosome spreads and indirect immunofluorescence of spread nuclei were performed as described previously except spheroplasting was done at 25 °C for 30 min [41]. To assess the level of cohesin association per chromosome, the average pixel intensity for each chromosome mass was determined. The background pixel intensity for each slide was determined by measuring the average pixel intensity for areas similar in size to spread nuclei. Subtracting the background intensity from each chromosome mass gave relative pixel intensity. At least 100 nuclei were analyzed per slide to generate an average relative pixel intensity per chromosome mass. To assay the stability of cohesin association with chromosomes, nuclei were spread on multiple slides in the absence of fixative with 0.25% Triton X-100 in PHEM buffer (60 mM Pipes, 25 mM Hepes, [pH 6.95], 10 mM EGTA, and 4 mM MgCl_2_) and incubated for 10 min. Each slide was then placed in a single coplin jar containing ∼50 ml 0.25% Triton X-100 in PHEM buffer with varying amounts of KCl for 30 min, while gently shaking. Following this incubation, nuclei were fixed by 4% PFA with 0.25% Triton X-100 in PHEM buffer, as before. Immunofluorescence was performed as above.

## Supporting Information

Figure S1 Colony Growth of Cells Expressing Dominant Negative *smc1* Alleles Cultures of 1377A1-4B (*SMC1,* odd numbers) and 1360–7C (*smc1–2,* even numbers) cells containing minichromosomes expressing the indicated *Smc1* allele were grown in SC-URA-LA media at 23 °C. Cultures were diluted to an identical cell density, and 5-fold serial dilutions were grown on either SC-URA-LA (uninduced) or SC-URA-LAG (induced) plates at (A) 23 °C or (B) 37 °C for 3–5 d.
(C) Expression of dominant negative *smc1* alleles. Protein extracts were prepared from the strain 1,377 A1-4B (*SMC1 MCD1-HA*) containing minichromosomes expressing the indicated *Smc1* allele. Cultures were grown as in Figure 3B and 3C.
(490 KB PDF)

Figure S2 Analysis of Cohesin Binding at Scc2/Scc4 Chromatin-Binding Sites Cultures of YMM11 (smc1–2 MCD1-HA) cells containing minichromosomes with different *Smc1* alleles were grown and arrested in metaphase (see Figure 4C legend). Cells were processed for ChIP (see Materials and Methods). Mcd1-HA binding at specific chromosomal loci was analyzed by PCR. Two Scc2/Scc4 binding sites on Chromosome VI (SGD 75200–76000 and SGD 220000–226000) were tested for Mcd1-6HA binding [42]. A representative dataset is shown for the SGD 75200–76000 region.
(45 KB PDF)

Table S1 Strain Table (20 KB DOC)

## Abbreviations

CAR: cohesin-associated region
ChIP: chromatin immunoprecipitation
HU: hydroxyurea
RID: random insertion dominant negative
Smc: structural maintenance of chromosomes
