# Supplementary material for: A Multi-Step Pathway for the Establishment of Sister Chromatid Cohesion
Source: PLoS Genet. 2007 Jan 19;3(1):e12. doi: 10.1371/journal.pgen.0030012 (PMC1779304; doi:10.1371/journal.pgen.0030012)
Supplement: Supplementary file 1 [file pgen.0030012.sg001.pdf]

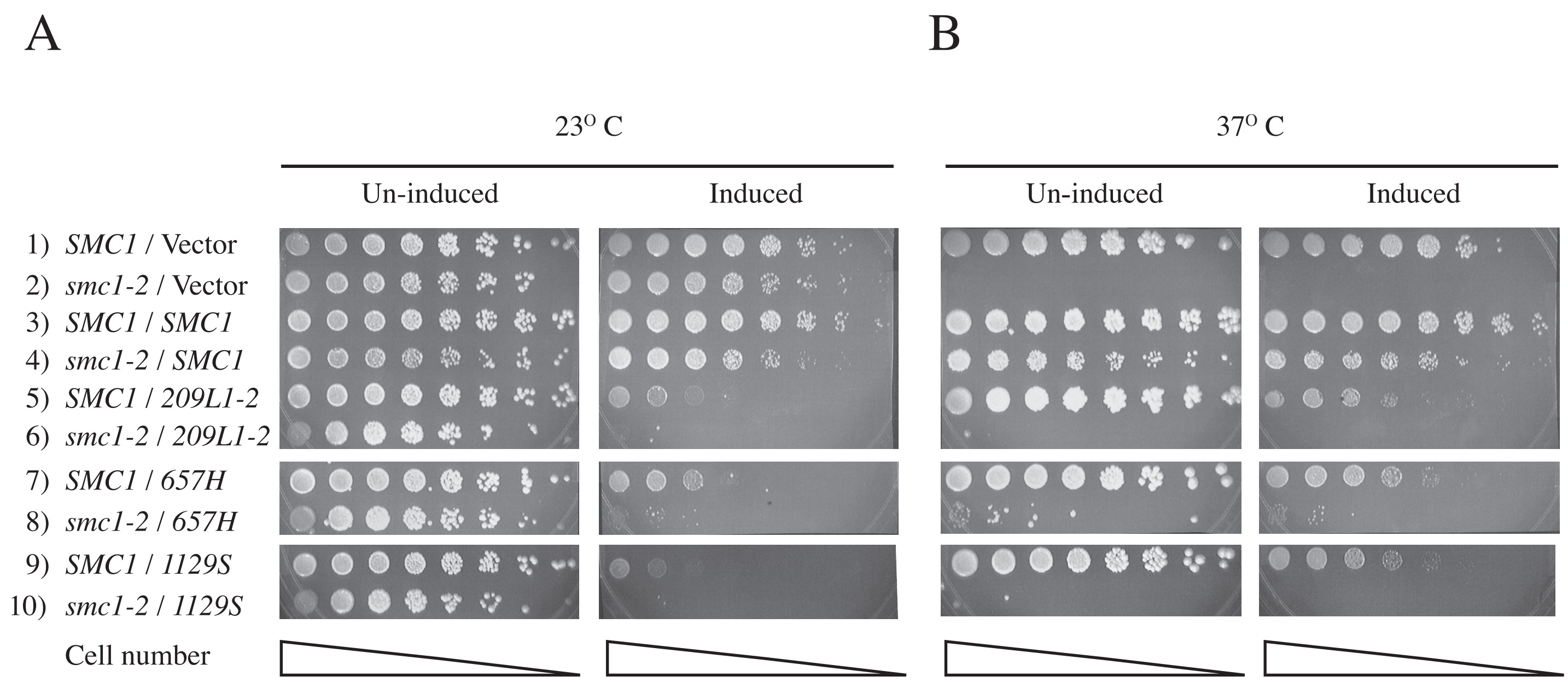

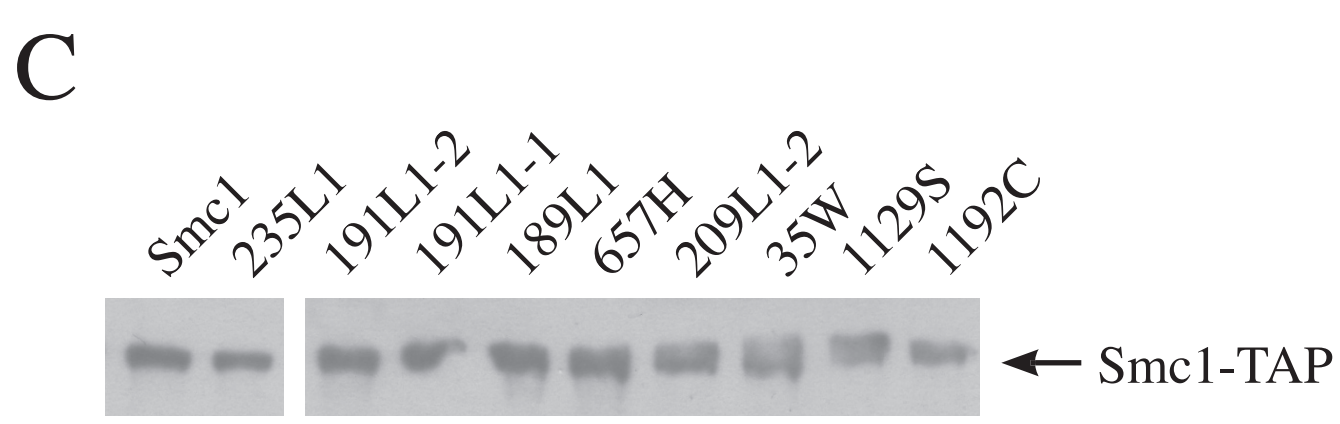

Supplemental Figure S1
