# Supplementary material for: A Multi-Step Pathway for the Establishment of Sister Chromatid Cohesion
Source: PLoS Genet. 2007 Jan 19;3(1):e12. doi: 10.1371/journal.pgen.0030012 (PMC1779304; doi:10.1371/journal.pgen.0030012)
Supplement: Supplementary file 2 [file pgen.0030012.sg002.pdf]

Smc1 | vector only | 657H | 209L1-2

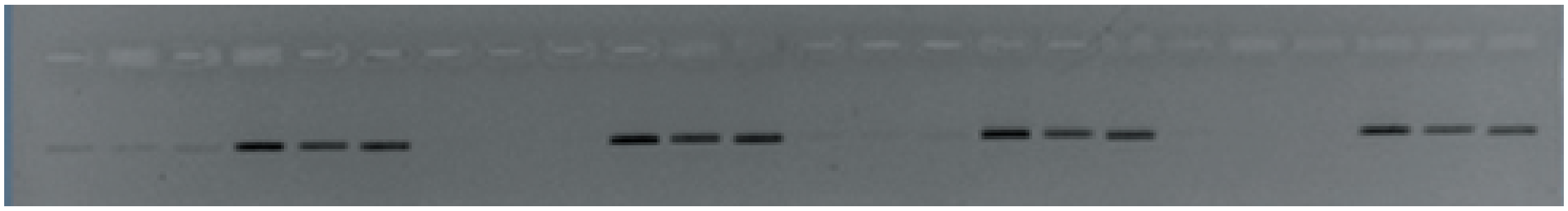

anti-Mcd1 IP | Input (1:100) | anti-Mcd1 IP | Input (1:100) | anti-Mcd1 IP | Input (1:100) | anti-Mcd1 IP | Input (1:100)

Supplemental Figure S2
