# Supplementary material for: A Multi-Step Pathway for the Establishment of Sister Chromatid Cohesion
Source: PLoS Genet. 2007 Jan 19;3(1):e12. doi: 10.1371/journal.pgen.0030012 (PMC1779304; doi:10.1371/journal.pgen.0030012)
Supplement: Supplementary file 3 [file pgen.0030012.st001.doc]

| Strain | Genotype |
| --- | --- |
| YMM1/pMM26 | *Mata SMC1::His5+ ura3-52 leu2Δ1 trp1Δ63 his3Δ200 lys2-801amber ade2-101ochre / CEN6 TRP1 pSMC1-SMC1-TAP Kan Amp* |
| YMM1/pMM26/AMH4 | *Mata SMC1::His5+ ura3-52 leu2Δ1 trp1Δ63 his3Δ200 lys2-801amber ade2-101ochre / CEN6 TRP1 pSMC1-SMC1-TAP Kan Amp / CEN6 URA3 pGAL1-SMC1-TAP Amp* |
| YMM-202 | *Mata smc1-2 ura3 LacI:KAN CHRIV::LacO-CLONNAT* |
| 1377A1-4B | *Mata MCD1-6HA ura3 leu2* |
| 2258G | *Mata/Matα SMC3-6MYC-HIS5/+ trp1/trp1 leu2/leu2 ura3/ura3 his3Δ200/his3Δ200* |
| YMM10 | *Mata ura3 bar1 smc1-2:LEU2* |
| YMM11 | *Mata MCD1-6HA ura3 bar1 smc1-2:LEU2* |
| YMM20 | *Mata PDS5-6HA ura3 bar1 smc1-2:LEU2* |
| YBS573 | *Mata ade2 ura3 HIS3::CTF7 LEU2:CTF7 MCD1-6HA* |
| YBS576 | *Mata ade2 ura3 HIS3::CTF7 LEU2:ctf7-203 MCD1-6HA* |
| 1360-7C | *Matα ura3 bar1 leu2 smc1-2:LEU2* |
